# A propensity score-matched analysis on the impact of patient and surgical factors on early periprosthetic joint infection in minimally invasive anterolateral and transgluteal total hip arthroplasty

**DOI:** 10.1007/s00402-022-04756-z

**Published:** 2023-01-11

**Authors:** Matthias Luger, Marcel de Vries, Sandra Feldler, Günter Hipmair, Tobias Gotterbarm, Antonio Klasan

**Affiliations:** 1 Department for Orthopedics and Traumatology, Kepler University Hospital GmbH, Krankenhausstrasse 9, 4020 Linz, Austria; 2 Johannes Kepler University Linz, Altenberger Strasse 69, 4040 Linz, Austria

**Keywords:** Minimally invasive, Total hip arthroplasty, Cementless, Anterolateral approach, Transgluteal approach, Periprosthetic joint infection

## Abstract

**Introduction:**

Increased risk of periprosthetic joint infection (PJI) in minimally invasive (MIS) total hip arthroplasty (THA) is still debated. This study aimed to identify differences in surgical and patient-related risk factors for PJI between an MIS anterolateral approach and transgluteal-modified Hardinge approach.

**Methods:**

A retrospective cohort of 5315 THAs performed between 2006 and 2019 at a single institution was screened. Short stem THAs performed via an MIS anterolateral approach in the supine position and standard straight stem THAs performed via a transgluteal modified Hardinge approach were included. Propensity score matching was performed to control for selection bias. After matching, 1405 (34.3%) short stem THAs implanted via MIS anterolateral approach and 2687 (65.7%) straight stem THAs implanted via a transgluteal modified Hardinge approach were included. The risk of PJI due to patient-specific and surgical factors was retrospectively analyzed using chi-square test and multivariate regression analysis.

**Results:**

PJI occurred in 1.1% in both MIS anterolateral and transgluteal approach (*p* = 0.823). Multivariate regression showed an increased infection risk for patients with a BMI between 35 and 39.99 kg/m^2^ (OR 6.696; CI 1.799–24.923; *p* = 0.005), which could not be demonstrated for transgluteal approach (OR 0.900; CI 0.900–4.144; *p* = 0.93). A BMI ≥ 40 kg/m^2^ (OR 14.150; CI 2.416–82.879; *p* = 0.003) was detected as a risk factor for PJI only in anterolateral approach. Increased operation time ≥ 121 min showed a significantly increased risk for PJI in the general cohort (OR 6.989; CI1.286–37.972; *p* = 0.024).

**Conclusion:**

Minimally invasive anterolateral and transgluteal THA show a comparable rate of early PJI within the first year of index surgery. A BMI of ≥ 35 kg/m^2^ was detected as a clear risk factor for infection in the anterolateral approach. Prolonged operation time ≥ 121 min increases the risk of PJI regardless of approach.

## Introduction

Total hip arthroplasty (THA) is one of the most successful surgeries in orthopedics, providing pain reduction, good functional outcomes, and improvement in quality of life [1, 2]. Although complication rates in THA are relatively low, periprosthetic joint infection (PJI) is a devastating complication, that can lead to revision surgery with increased morbidity and mortality [3–5].

Several patient-specific factors such as obesity [6–12], diabetes [6, 12–14], rheumatoid arthritis [8, 12], alcohol abuse [12] and smoking status [15] are considered as potential risk factors for postoperative wound complications and PJI after total joint arthroplasty (TJA). Apart from patient-specific aspects, various surgical factors seem to be related to an increased risk of PJI [8].

In recent years, minimally invasive surgical (MIS) anterior-based approaches have gained popularity because they are associated with faster postoperative rehabilitation, less pain, and better functional outcomes than conventional surgical approaches [2, 16]. One of these MIS approaches is the MIS anterolateral approach. The risk of complications in the MIS anterolateral approach and in particular of the risk for PJI increases significantly in severely and morbidly obese patients [7]. In a big registry study, Smith et al. [8] found an increased PJI revision rate by about 1.6-fold when compared to the posterior approach. In contrast, Sheth et al. [17], do not report a significantly increased risk for surgical complications and especially septic revision in the anterolateral approach compared to the posterior approach. The rate of septic revision was reported of being two times higher in the direct lateral approach (DLA) (0.5% vs. 1.1%) with a hazard ratio of 2.15 for DLA compared to 0.98 for the anterolateral approach, however without statistical significance [17]. In a recent meta-analysis by Acuña et al. [18] did not find a significantly increased risk for PJI in the anterolateral approach when compared to the direct anterior approach (DAA). However, data about the incidence of PJI and potential risk factors for infection after THA via MIS anterolateral approach compared to conventional standard approaches are inconclusive. Therefore, the aim of this study was to identify risk factors and differences in risk for periprosthetic joint infection (PJI) in primary THA using a minimally invasive anterolateral cementless short stem THA and transgluteal cementless straight stem THA within 12 months after index surgery.

## Patients and methods

The institutional electronic database was used to obtain information on patients who underwent THA between 2006 and 2019. In total, 5315 THAs in 5205 patients have been performed in this period. Inclusion criteria were defined as cementless short stem THA via a minimally-invasive anterolateral approach in supine positioning [19] or cementless straight stem THA via a modified Hardinge approach [20]. Diagnosis for inclusion was primary osteoarthritis, avascular necrosis of the head and hip dysplasia. All forms of secondary osteoarthritis due to posttraumatic deformities or rheumatoid arthritis and all cases with previous surgeries on the affected side were excluded. Additionally, all forms of other approaches were excluded. Cemented THA, the use of deviating implants such as revision cups or stems were excluded. We retrospectively screened every case in this time period that was revised for any reason within the first year. In a second step, all revisions were screened if they met criteria for a periprosthetic joint infection (PJI). A PJI was defined according to the new scoring system from 2018 by Parvizi et al. [21]. As the relevant parameters for the minor criteria were not available in all cases, only PJIs could be included, that met one of the major criteria. Alpha-Defensin test was performed in selected cases. The cases, in which the alpha-defensin test was performed, also fulfilled the major criteria and therefore overruled the minor criteria.

Transgluteal approach was performed as the standard approach between the years 2006 and 2011 at the institution. In 2011 anterolateral approach was introduced at the institution. Between the years 2011 and 2015 MIS anterolateral approach was performed in parallel with the transgluteal approach. From the beginning of the year 2016 until the end of 2019 MIS anterolateral approach was performed as the standard approach. From 2016 transgluteal approach was only performed in selected cases by the preference of the performing surgeon. With transitioning from transgluteal to anterolateral approach as the standard approach for primary THA at our institution, also residents were primarily trained in the anterolateral approach from 2016 and onwards.

The study was approved by the institutional review board (EK-No.: 1194/2021). Because of the retrospective anonymized evaluation of pre-existing medical records, an informed consent was not required. All procedures performed in studies involving human participants were in accordance with the ethical standards of the institutional and/or national research committee and with the 1964 Helsinki declaration and its later amendments or comparable ethical standards.

### Surgical technique

In total 28 surgeons performed the surgeries. The surgeries were performed by 8 consultants and 10 residents. 10 surgeons performed the surgeries as residents and consultants. All surgeons performed the surgeries in a standardized manner and were partly or fully trained at the authors’ institution. The institutional transition from transgluteal straight stem THA to MIS anterolateral short stem THA was introduced by two experienced consultants. After gaining enough experience, the transition was then extended to further surgeons in the team under the supervision of these two consultants. Operation time was defined as the time in minutes from skin incision to skin closure.

All surgeries were performed under laminar airflow. Extremity preparation was performed with threefold antiseptic scrub with alcohol disinfectant in all cases. Routinely draping with sterile adhesive surgical iodine film was used only by a certain number of surgeons. The standardized peri- and postoperative protocol was identical in all cases, including single-shot antibiotics (Cefuroxime 1.5 g i.v. directly pre-operatively), Indomethacin 75 mg twice daily for the prevention of heterotopic ossification on day one to four post-operatively, and 40 mg low-molecular-weight heparin or Rivaroxaban 10 mg for 28 days post-operatively as venous thromboembolic event prophylaxis.

Minimally invasive anterolateral approach was performed in supine positioning. A skin incision was centered over the greater trochanter. An incision at the border between the tensor fasciae latae and the tractus iliotibilias was performed. Then, the Watson-Jones interval between tensor fasciae latae and gluteus medius was bluntly dissected. A capsulectomy was performed in each case. Full weight-bearing was allowed immediately on the day of surgery. Drainage was used until the end of 2017 in every case in an anterolateral approach. In 2018 drainage was used only to the surgeon’s preference. From 2019 drainage was not used routinely in the anterolateral approach.

The direct lateral approach (DLA) by Hardinge was first described in 1982 [22]. The modified Hardinge approach has previously been described by Frndak et al. [20]. The modified Hardinge approach was performed with the positioning the patient in supine positioning. A lateral skin incision was used centered over the greater trochanter. Access to the hip joint was gained through an abductor muscle split approach. The fibers of the gluteus medius were split longitudinally at the junction of the anterior third to posterior two-thirds of the muscle belly. The gluteus minimus and capsule were then divided vertically along the same incision parallel to the gluteus medius split. Then a capsulectomy of the anterior capsule was performed. Full weight-bearing was allowed on day one after surgery. Drainage was used in every case in transgluteal approach.

Suturing was done either by skin clamping or intracutaneous suturing. Intracutaneous suturing was the standard wound closure until 2019. Skin clamping was the standard wound closure from the beginning of 2019. The sutures were removed by the family practitioner or by the rehabilitation staff or in certain cases at the outpatient department of the institution after 12–14 days of surgery. Patients were informed at dismissal by the medical report and orally to readmit at the institution in case of any signs PJI. Follow-up was scheduled at 3 months and 1 year postoperatively.

### Implants

In a minimally invasive anterolateral approach, a cementless, curved short stem (Fitmore^®^ stem, Zimmer Biomet, Warsaw, IN, USA) was digitally templated using mediCAD^®^ version 5.1 (Hectec GmbH, Altdorf, Germany). Fitmore^®^ hip stem is a titanium alloy stem (Ti Al6V4) that has a porolock Ti-VPS coating in the proximal part to enhance bone ingrowth and is available in four different neck angle options (127°, 129°, 137°, 140°). A cementless titanium press-fit cup with or without screws (Allofit^®^/-S, Zimmer Biomet, Warsaw, IN, USA) or two types of cementless threaded cups (Alloclassic CSF^®^/ Alloclassic Variall^®^, both Zimmer Biomet, Warsaw, IN, USA) were used. In the transgluteal approach a cementless Zweymüller straight stem in two variations was used (Alloclassic SL/SLO; Alloclassic SLV; both Zimmer Biomet, Warsaw, IN, USA). In the transgluteal approach, a cementless titanium press-fit cup with or without screws (Allofit^®^/-S/IT, Zimmer Biomet, Warsaw, IN, USA) or two types of cementless threaded cups (Alloclassic CSF^®^/ Alloclassic Variall^®^, both Zimmer Biomet, Warsaw, IN, USA) were used. Independent from the approach, highly cross-linked polyethylene liners (Alpha Durasul^®^, Gamma Durasul^®^, Alloclassic CSF Durasul^®^, Longevity IT Liner^®^, all Zimmer Biomet, Warsaw, IN, USA) were used in every case. As femoral heads two types of ceramic heads were used (BIOLOX forte, CeramTec GmbH, DE; Sulox, Zimmer Biomet, Warsaw, IN, USA) as well as Cobalt-chrome (CoCr) metal heads (Durasul CoCr, Zimmer Biomet, Warsaw, IN, USA).

### Statistics

Descriptive analysis was performed for patient demographics. A Shapiro–Wilk test for normality was performed to determine whether continuous data were normally distributed. As the variables were normally distributed, a Pearson’s chi-square test was performed for categorical variables and a student’s *t* test was performed for continuous variables. Because of statistically significant differences in the patient demographics a propensity score matching was performed using the caliper technique. The caliper was set at 0.2. The propensity score matching was performed for patient age at operation, Body Mass Index (BMI; kg/m^2^) ASA Score (American Society of Anesthesiologists Score), gender, diagnosis, operation side, smoking status, alcohol consumption, diabetes and the surgical factors approach, operation time and the surgeon’s experience. The risk of PJI was calculated for all patient and surgical factors that were included in the propensity score matching. A post hoc power analysis was performed. With the total sample size of 4092 patients, an alpha of 0.05 and an omega (*ω*) of 0.003, a power (beta) of 0.54 was calculated. The rates of revision due to PJI were recorded for all patients and divided by approach. A multivariate regression model was calculated for all patient and surgical factors on the risk of PJI for the general cohort. Additionally, a multivariate regression model was calculated and divided by approach. All significant factors of the univariate analysis were then used for multivariate regression analysis. Data were analyzed using SPSS version 28 (IBM SPSS statistics, Chicago, IL, USA).

## Results

### Propensity score matching

**Fig. 1:**
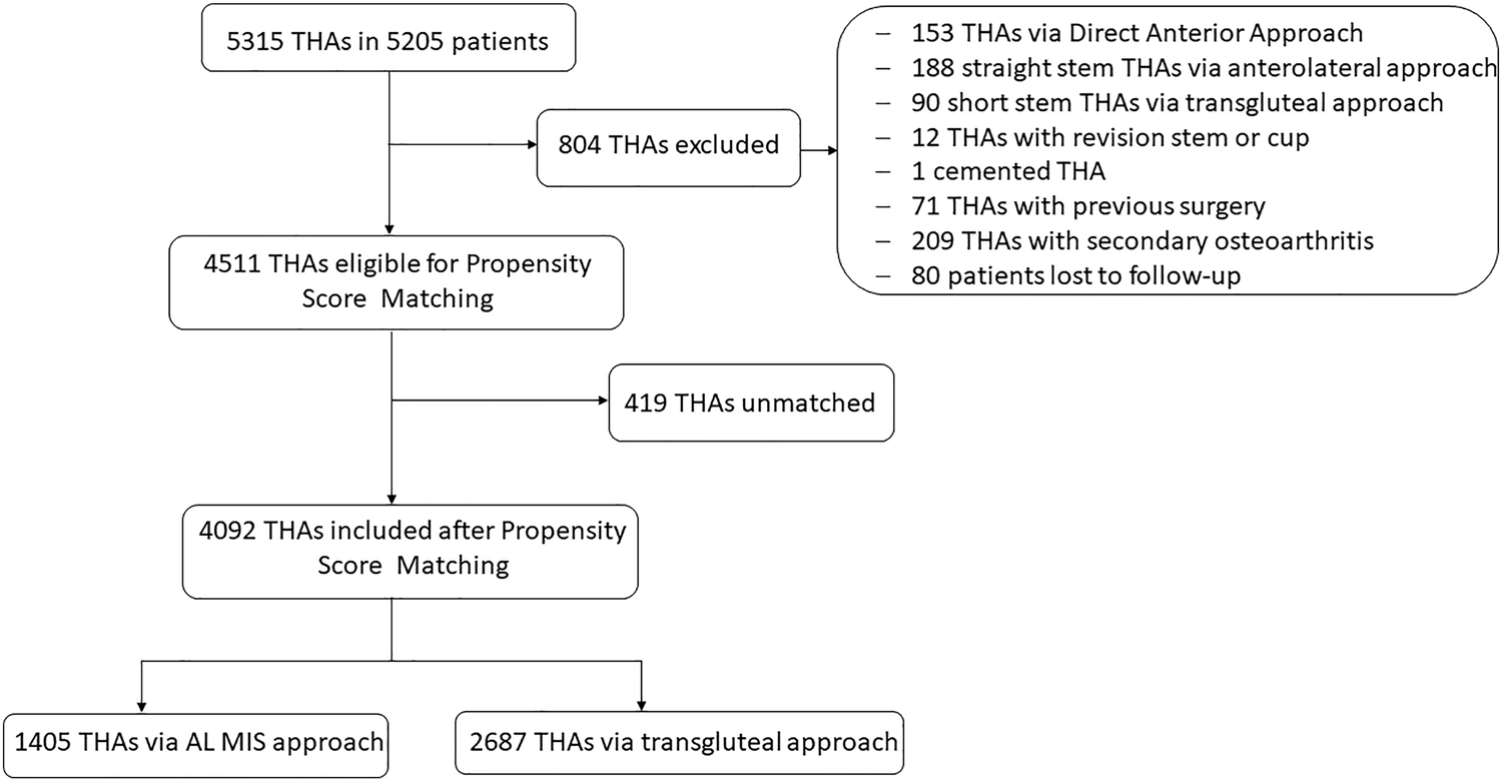
Consort Diagram for inclusion and exclusion of patients

In total, 4511 THAs have met the inclusion criteria included in this study. A total of 806 THAs did not meet the inclusion criteria, Fig. 1. Of these 806 patients, 80 patients have been lost to follow-up. Both groups differed significantly in the patient age at operation (*p* = 0.011), experience of the surgeon (< 0.001), operation time (< 0.001), ASA Score (*p* < 0.001) and smoking status (0.046), Table 1. By propensity score matching 409 patients were excluded. Therefore, 4092 THAs were included after the propensity score matching in the final analysis. The patient demographics did not differ between both approaches after matching in all categories, Table 1. In 1405 cases, short stem THA (34.3%) was performed via a minimally-invasive anterolateral approach and 2687 straight stem THAs (65.7%) were implanted via a transgluteal modified Hardinge approach.

**Table 1:** Patient demographics

	Pre-matched cohort			Post-matched cohort		
	Anterolateral	Lateral		Anterolateral	Lateral	
	Mean (± SD)	Mean (± SD)	*P* value	Mean (± SD)	Mean (± SD)	*P* value
Number of patients	1410 (31.3%)	3101 (68.7%)		1405 (34,3%)	2687 (65,7%)	
Age at operation	67.18 (± 11.55)	66.29 (± 12.07)	**0.011**	67.15 (± 11.56)	66.81 (± 11.96)	0.383
BMI	27.9 (± 4.8)	28 (± 4.74)	0.527	27.92 (± 4.8)	27.77 (± 4.5)	0.318
Sex			0.343			0.606
Female	783 (55.5%)	1675 (54%)		780 (55.5%)	1469 (54.7%)	
Male	627 (44.5%)	1426 (46%)		625 (44.5%)	1218 (45.3%)	
Diagnosis			0.077			0.168
Primary OA	1177 (83.5%)	2501 (80.7%)		1172 (83.4%)	2177 (81.1%)	
AVN	146 (10.3%)	276 (12.1%)		146 (10.4%)	321 (11.9%)	
Hip Dyplasia	87 (6.2%)	224 (7.2%)		87 (6.2%)	189 (7%)	
Surgeon’s experience			** < 0.001**			0.060
Consultant	1128 (80%)	2155 (69.5%)		1123 (79.9%)	2079 (77.4%)	
Resident	282 (20%)	946 (30.5%)		282 (20.1%)	608 (22.6%)	
Operation time	80.29 (± 23.32)	84.73 (± 22.76)	** < 0.001**	80.38 (± 23.3)	81.75 (± 20.25)	0.052
Side			0.646			0.522
Left	658 (46.7%)	1470 (47.4%)		654 (46.5%)	1279 (47.6%)	
Right	752 (53.3%)	1631 (52.6%)		751 (53.5%)	1408 (52.4%)	
ASA			** < 0.001**			0.146
1	276 (19.6%)	494 (15.9%)		272 (19.4%)	469 (17.5%)	
2	823 (58.4%)	2011 (64.9%)		823 (58.6%)	1673 (62.3%)	
3	305 (21.6%)	581 (18.7%)		304 (21.6%)	533 (19.8%)	
4	6 (0.4%)	15 (0.5%)		6 (0.4%)	12 (0.4%)	
Diabetes	176 (12.5%)	385 (12.4%)	0.950	176 (12.5%)	323 (12%)	0.639
Smoking	240 (17%)	456 (14.7%)	**0.046**	235 (16.7%)	410 (15.3%)	0.221
Alcohol	300 (21.3%)	645 (20.8%)	0.715	299 (21.3%)	557 (20.7%)	0.680

Bold letters indicate significant values

*BMI* Body Mass Index kg/m^2^, *primary OA* primary osteoarthritis, *AVN* avascular necrosis of the femoral head, *ASA* American Society of Anesthesiologists

### Comparison between both approaches

In total, 45 PJIs (1.1%) were detected within 12 months of index surgery. All cases met the major criteria by Parvizi et al. [21]. In all cases, either two positive cultures of the same organism or a sinus tract with evidence of communication to the joint or visualization of the prosthesis or both were documented. Rate of PJI was 1.1% in the anterolateral approach compared to 1.1% in the transgluteal approach (*p* = 0.862), Table 2. The number of infections and the testing for the occurrence of PJI in the general cohort are shown in Table 2. Increased BMI was statistically significant in the general cohort (*p* = 0.022), Table 2. In the anterolateral approach, the number of infections were significantly higher in patients with increased BMI (*p* < 0.001), Table 3. In the transgluteal approach the number of infections were significantly higher in patients with increased ASA Score (*p* = 0.034) and in diabetic patients (*p* = 0.044), Table 4.

**Table 2:** Chi-Square test for PJI and patient or surgical factors for the general cohort

	Total (*n*)	Infection (*n*)	Infection (%)	*P* value
Approach				0.862
Anterolateral	1405	16	1.1	
Transgluteal	2687	29	1.1	
BMI (kg/m^2^)				**0.022**
< 35	3792	37	1.0	
35–40	240	6	2.5	
> 40	60	2	3.3	
ASA				0.087
1	741	6	0.8	
2	2496	23	0.9	
3	837	16	1.9	
4	18	0	0.0	
Gender				0.084
Female	2249	19	0.8	
Male	1843	26	1.4	
Age (years)				0.059
< 60	1112	6	0.5	
60–69	1138	11	1.0	
70–79	1318	22	1.7	
≥ 80	512	6	1.2	
Diagnosis				0.222
Primary OA	3349	36	1.1	
AVN	467	8	1.7	
Hip dysplasia	1	1	0.4	
Side				0.706
Left	1933	20	1.0	
Right	2159	25	1.2	
Smoking				0.709
Yes	645	8	1.2	
No	3447	37	1.1	
Alcohol				0.559
Yes	856	11	1.3	
No	3236	34	1.1	
Diabetes				0.108
Yes	499	9	1.8	
No	3593	36	1.0	
Operation time (min)				0.095
≤ 60	472	2	0.4	
61–90	2550	26	1.0	
91–120	870	12	1.4	
≥ 121	200	5	2.5	
Experience				0.775
Consultant	3202	36	1.1	
Resident	890	9	1.0	

Bold letters indicate significant values

*BMI* Body Mass Index kg/m^2^, *primary OA* primary osteoarthritis, *AVN* avascular necrosis of the femoral head, *ASA* American Society of Anesthesiologists

**Table 3:** Chi-Square test for PJI and patient or surgical factors separated for anterolateral approach

	Total (*n*)	Infection (*n*)	Infection (%)	*P* value
BMI (kg/m^2^)				** < 0.001**
< 35	1282	10	0.8	
35–40	99	4	4.0	
> 40	24	2	8.3	
ASA				0.899
1	272	2	0.7	
2	823	10	1.2	
3	304	4	1.3	
4	6	0	0.0	
Gender				0.655
Female	780	8	1.0	
Male	625	8	1.3	
Age (years)				0.421
< 60	208	1	0.5	
60–69	864	9	1.0	
70–79	258	4	1.6	
≥ 80	75	2	2.7	
Diagnosis				0.350
Primary OA	1172	13	1.1	
AVN	146	3	2.1	
Hip dysplasia	87	0	0.0	
Side				0.821
Left	654	7	1.1	
Right	751	9	1.2	
Smoking				0.259
Yes	1170	15	1.3	
No	235	1	0.4	
Alcohol				0.388
Yes	299	2	0.7	
No	1106	14	1.3	
Diabetes				0.997
Yes	176	2	1.1	
No	1229	14	1.1	
Operation time (min)				0.319
≤ 60	373	1	0.3	
61–90	401	6	1.5	
91–120	467	7	1.5	
≥ 121	164	2	1.2	
Experience				0.262
Consultant	1123	11	1.1	
Resident	282	5	1.8	

Bold letters indicate significant values

*BMI* Body Mass Index kg/m^2^, *primary OA* primary osteoarthritis, *AVN* avascular necrosis of the femoral head, *ASA* American Society of Anesthesiologists

**Table 4:** Chi-Square test for PJI and patient or surgical factors separated for transgluteal approach

	Total (*n*)	Infection (*n*)	Infection (%)	*P* value
BMI (kg/m^2^)				0.761
< 35	2510	27	1.1	
35–40	141	2	1.4	
> 40	36	0	0.0	
ASA				**0.034**
1	469	4	0.9	
2	1673	13	0.8	
3	533	12	2.3	
4	12	0	0.0	
Gender				0.069
Female	1469	11	0.7	
Male	1218	18	1.5	
Age (years)				0.110
< 60	749	5	0.7	
60–69	737	5	0.7	
70–79	851	15	1.8	
≥ 80	250	4	1.1	
Diagnosis				0.539
Primary OA	2177	23	1.1	
AVN	321	5	1.6	
Hip dysplasia	189	1	0.5	
Side				0.764
Left	1279	13	1.0	
Right	1408	16	1.1	
Smoking				0.181
Yes	410	7	1.7	
No	2277	22	1.0	
Alcohol				0.169
Yes	2130	9	0.9	
No	557	20	1.6	
Diabetes				**0.044**
Yes	323	7	2.2	
No	2364	22	0.9	
Operation time (min)				0.304
≤ 60	264	1	0.4	
61–90	1686	17	1.0	
91–120	612	8	1.3	
≥ 121	125	3	2.4	
Experience				0.253
Consultant	2079	25	1.2	
Resident	608	4	0.7	

Bold letters indicate significant values

*BMI* Body Mass Index kg/m^2^, *primary OA* primary osteoarthritis, *AVN* avascular necrosis of the femoral head, *ASA* American Society of Anesthesiologists

### Regression analysis

The multivariate regression analysis for the general cohort and separated by approach is shown in detail in Table 5. Multivariate regression analysis showed a significantly increased odds ratio (OR) for PJI in the total study group in patients between 70 and 79 years at operation (OR 4.687; CI 1.629–14.536), Table 5. The OR was also increased in patients 80 years of age at operation or older (OR 3.723; CI 0.955–14.522) but without statistical significance (*p* = 0.059), Table 5. The OR for increasing operation time increased throughout all groups but only showed a statistically increased risk in THAs with ≥ 121 min of operation time (OR 6.989; CI 1.286–37.972), Table 5.

**Table 5:** Multivariate analysis for the risk of PJI for patient or surgical factors for all patients and divided by approach

	All		Anterolateral		Transgluteal	
	OR (CI)	*P* value	OR (CI)	*P* value	OR (CI)	*P* value
BMI (kg/m^2^)						
< 35	1.000	–	1.000	–	1.000	–
35–40	2.486 (0.993–6.223)	0.052	6.696 (1.799–24.923)	**0.005**	0.900 (0.900–4.144)	0.93
> 40	2.851 (0.621–13.089)	0.178	14.150 (2.416–82.879)	**0.003**	NV	–
ASA						
1	1.000	–	1.000	–	1.000	–
2	0.758 (0.268–2.144)	0.602	0.754 (0.145–3.931)	0.738	0.614 (0.173–2.178)	0.451
3	1.056 (0.331–3.368)	0.926	0.650 (0.090–4.485)	0.650	1.345 (0.333–5.430)	0.678
4	NV	–	NV	–	NV	–
Gender						
Female	1.000	–	1.000	–	1.000	–
Male	1.694 (0.893–3.215)	0.106	1.624 (0.559–4.179)	0.373	1.787 (0.781–4.089)	0.169
Age (years)						
< 60	1.000	–	1.000	–	1.000	–
60–69	2.291 (0.765–6.964)	0.139	5.822 (0.622–54.467)	–	1.462 (0.365–5.856)	0.592
70–79	4.687 (1.629–14.536)	**0.005**	8.851 (0.867–90.327)	0.066	4.404 (1.206–16.085)	**0.025**
≥ 80	3.723 (0.955–14.522)	0.058	8.484 (0.556–129.393)	0.124	3.240 (0.626–16.755)	0.161
Diagnosis						
Primary OA	1.000	–	1.000	–	1.000	–
AVN	1.490 (0.670–3.313)	0.328	1.915 (0.495–7.411)	0.347	1.239 (0.446–3.441)	0.763
Hip dysplasia	0.741 (0.087–5.672)	0.741	NV	–	0.829 (0.093–7.405)	0.867
Side						
Left	1.000	–	1.000	–	1.000	–
Right	1.165 (0.640–2.119)	0.617	1.181 (0.423–3.299)	0.751	1.141 (0.538–2.418)	0.731
Smoking						
No	1.000	–	1.000	**–**	1.000	–
Yes	1.869 (0.803–4.350)	0.147	0.526 (0.062–4.466)	0.556	3.023 (1.126–8.119)	**0.028**
Alcohol						
No	1.000	–	1.000	**–**	1.000	–
Yes	1.054 (0.509–2.183)	0.887	0.505 (0.062–4.466)	0.526	1.433 (0.579–3.442)	0.388
Diabetes						
No	1.000	–	1.000	**–**	1.000	–
Yes	1.295 (0.590–2.841)	0.519	0.530 (0.107–2.634)	0.438	1.897 (0.748–4.816)	0.178
Operation time (min)						
≤ 60	1.000	–	1.000	–	1.000	–
61–90	2.581 (0.607–10.980)	0.199	2.232 (0.272–18.299)	0.454	3.049 (0.400–23.224)	0.272
91–120	3.663 (0.794–16.909)	0.096	2.414 (0.240–24.236)	0.454	4.304 (0.522–35.466)	0.175
≥ 121	6.989 (1.286–37.972)	**0.024**	6.856 (0.517–90.862)	0.144	9.318 (0.906–95.817)	0.061
Experience						
Consultant	1.000	–	1.000	–	1.000	–
Resident	0.719 (0.339–1.528)	0.392	2.068 (0.663–6.449)	0.210	0.389 (0.132–1.150)	0.088

Bold letters indicate significant values

*BMI* Body Mass Index kg/m^2^, *primary OA* primary osteoarthritis, *AVN* avascular necrosis of the femoral head, *ASA* American Society of Anesthesiologists

The multivariate analysis separated by approach showed a significantly increased risk for PJI in the anterolateral approach for patients with a BMI ≥ 35–39.99 kg/m^2^ (OR 6.696; CI 1.799–24.923) and BMI ≥ 40 kg/m^2^ (OR 14.150; CI 2.416–82.879), Table 5. In the transgluteal approach, a patient aged between 70 and 79 years at operation (OR 4.404; CI 1.206–16.085) and smoking (OR 3.023; CI 1.126–8.119) were identified as independent risk factors for PJI.

## Discussion

In the current study, we retrospectively analyzed the rates of periprosthetic joint infection within 12 months from index surgery in propensity-score matched cohorts including minimally invasive anterolateral short stem THA and transgluteal straight stem THA and evaluated potential risk factors for infection. We did not find a statistically significant difference in the rates of PJI within the first year of index surgery between an anterolateral MIS approach and a transgluteal Hardinge approach, while the risk for occurrence of a PJI was significantly higher in severely obese patients in an anterolateral approach and increased surgical time longer than 120 min was a significantly increased risk factor in both approaches.

The incidence of PJI is reported within a range of 0.3–3% [9, 23]. We found comparable PJI rates of 1.1% in both groups (*p* = 0.862). Ilchmann et al. [24] found a rate of PJI of 1.7% for DLA. Shohat et al. [25] reported a rate of 1.3% in DLA. Some authors suggest higher rates of PJI and numbers of revision surgeries due to PJI with MIS approaches [8, 26]. Smith et al. [8] reported THAs implanted via an anterolateral approach at a higher risk of revision for postoperative infection compared to the posterior approach (PA) (OR 1.61; CI 1.16–2.23; *p* = 0.005). Other studies based on nationwide registries could not find a negative influence of MIS approaches on the risk of revision due to infection [27–29]. Sheth et al. [17] could not report a statistically significant increased risk for septic revision in an anterolateral approach, with a reduced rate of early dislocation, concluding to be the main advantage of an anterolateral approach. We also report comparable rates of PJI in anterolateral and transgluteal approach. Additionally, the low rate of dislocation of an MIS anterolateral approach and a cementless short stem has been previously [7]. Therefore, an anterolateral MIS approach might be favorable due to the reduced rate of early complications without leading to an increased rate of PJI compared to a standard transgluteal approach.

Although the overall infection rate was equivalent in both cohorts, the anterolateral approach was associated with higher infection rates as BMI increased. Severely (BMI ≥ 35 kg/m^2^) and morbidly obese patients (BMI ≥ 40 kg/m^2^) receiving THA via anterolateral approach were at a higher risk of developing PJI than obese patients in the transgluteal approach group. Obesity has previously been demonstrated to increase the risk of postoperative wound complication, deep infection, and revision surgery due to infection in THA via MIS approaches [7, 8, 30, 31]. A recent systematic review by Shah et al. [32] did not find any significantly increased risk for PJI in the anterolateral approach. However, the used cut-off was a BMI of 30 kg/m^2^. In the present study, a BMI above 35 kg/m^2^ did not have a statistically significant impact on PJI rates in the transgluteal approach group. Therefore, the transgluteal approach might be favorable compared to the MIS anterolateral approach regarding the risk of early PJI in obese patients.

Prolonged surgical duration has previously been shown to increase the risk of surgical site infection in total joint arthroplasty [12, 33]. Every 20-min increase in operation time is related to an almost 25% higher risk of PJI in primary TJA [33]. In the current study, an operation time ≥ 121 min was identified as an independent risk factor for PJI. However, this increased risk was only significant in the general cohort. When separated by approach, the OR increased with longer operation times, but without statistical significance.

Diabetes is a well-known risk factor for the risk PJI in THA [8]. Jämsen et al. [6] report a more than twofold increase in PJI risk for patients diagnosed with diabetes (OR 2.31, CI 1.12–4.72), independent of BMI. Iorio et al. [34] found a four times higher risk of infection in patients with diabetes undergoing total hip or knee arthroplasty. In the present study, testing for significance revealed a significantly higher number of PJIs in patients diagnosed with diabetes in the transgluteal approach cohort. However, multivariate analysis did not show a significant influence of diabetes on the occurrence of PJI. Some studies suggest that the higher incidence of surgical site infections in patients diagnosed with diabetes might be limited to those with poorly controlled disease [35, 36]. Most THAs implanted via transgluteal approach were performed at the beginning of the study period before the transition from transgluteal to anterolateral as the standard approach at our institution. Possibly, antidiabetic treatment and, therefore, glycemic control in patients diagnosed with diabetes have improved over the study period. However, consistent data on preoperative glucose level and glycated hemoglobin were not available in the present retrospective study.

Limitations of this study mainly conclude the retrospective study design. Therefore, baseline differences could be found for age, experience of the surgeon, ASA score and smoking status between both study groups. To control for selection bias and to eliminate possible confounders, propensity score matching incorporating patient demographics, comorbidities and surgery-related variables was performed. However, the anterolateral and transgluteal approach were not performed concurrently over the study period as the standard approach transitioned from the transgluteal to MIS anterolateral approach. Additionally, the follow-up period was defined as 12 months after index surgery. However, the retrospective data analysis of our institutional electronic data does not provide reliable data for a longer follow-up period because of increasing patients lost-to-follow-up after 12 months. Because of the very long time period of included patients, we cannot provide full information and data to fulfill the minor criteria for the new scoring system from 2018 by Parvizi et al. [21]. Therefore, only PJIs that fulfilled the major criteria could be included, leading to the possibility of overseeing low-grade PJIs. Due to the retrospective study design, data on preoperative glucose levels or glycated hemoglobin cannot be presented consistently. Additionally, preoperative risk factors were handled individually by the operating surgeon. Therefore, we cannot give conclusive information on the different preoperative thresholds for operating diabetic patients or patients with elevation of inflammatory markers such as C-reactive protein as it was handled individually. Intraoperative differences between surgeons were also not recorded consistently such as the use of iodine film. However, as a strength, apart from the differences between surgeons, we report a very standardized study collective. Furthermore, we report a very large study cohort with clear inclusion and exclusion criteria. The number of patients is unequally distributed with fewer cases in the short-stem group. However, we controlled for this unequal distribution by performing the propensity score matching to reduce the risk for bias due to unequal group sizes. A further limitation of the study is the presentation of only one complication. As there may be an increased risk for PJI in certain groups, a minimally invasive approach might have the potential to lead to a decrease of other complications such as deep vein thrombosis or other medical complications because a faster mobilization might be feasible. Another limitation of the study is the high number of different surgeons and also the inclusion of training operations of residents. However, all surgeons performed the surgeries in a standardized manner and were partly or fully trained at the authors’ institution. The institutional transition from transgluteal straight stem THA to MIS anterolateral short stem THA was introduced by two experienced consultants. After gaining enough experience, the transition was then extended to further surgeons in the team under the supervision of these two consultants.

## Conclusion

Minimally invasive anterolateral and transgluteal THA show a comparable rate of early PJI within the first year of index surgery. A BMI of ≥ 35 kg/m^2^ was detected as a clear risk factor for infection in the anterolateral approach. Prolonged operation time ≥ 121 min increases the risk of PJI regardless of approach.

## Data Availability

Data and materials are available on request.

## Appendix

See Table

**Table 6:** Distribution of PJIs by the year of operation for all patients and separated by approach

	All		Anterolateral		Transgluteal	
Year	Total	Infections, *n* (%)	Total	Infections, *n* (%)	Total	Infections, *n* (%)
2006	234	3 (1.3)	0	0 (0.0)	234	3 (1.3)
2007	261	1 (0.4)	0	0 (0.0)	261	1 (0.4)
2008	252	0 (0.0)	0	0 (0.0)	252	0 (0.0)
2009	251	3 (1.2)	0	0 (0.0)	251	3 (1.2)
2010	313	3 (1.0)	0	0 (0.0)	313	3 (1.0)
2011	303	5 (1.7)	4	0 (0.0)	299	5 (1.7)
2012	302	1 (0.3)	10	0 (0.0)	292	1 (0.3)
2013	293	3 (1.0)	37	0 (0.0)	256	3 (1.2)
2014	307	2 (0.7)	117	0 (0.0)	190	2 (1.1)
2015	294	4 (1.4)	156	2 (1.3)	138	2 (1.4)
2016	309	3 (1.0)	216	2 (0.9)	93	1 (1.1)
2017	320	6 (1.9)	271	3 (1.1)	49	3 (6.1)
2018	327	9 (2.8)	295	8 (2.7)	32	1 (3.2)
2019	326	2 (0.6)	298	1 (0.3)	27	1 (3.7)

6

## Abbreviations

ASA Score: American Society of Anesthesiologists Score
BMI: Body Mass Index (kg/m^2^)
CI: Confidence interval
DAA: Direct anterior approach
DLA: Direct lateral approach
MIS approach: Minimally invasive surgical approach
OR: Odds Ratio
PA: Posterior approach
PJI: Periprosthetic joint infection
THA: Total hip arthroplasty
TJA: Total joint arthroplasty
